# Lactic acid bacterial strains isolated from *Attiéké* (semolina cassava): bio-tools for combating emergent pathogens in the context of climate change

**DOI:** 10.3389/fmicb.2026.1841085

**Published:** 2026-06-16

**Authors:** Wahauwouélé Hermann Coulibaly, Yabo Majoie Géroxie Tohoyessou, Avent Brice Messan Ohin, Ange Olivier Parfait Yao, Paul-Alexandru Popescu, Comlan Kintomagnimessè Celestin Tchekessi, Noël Sènou Tovide, Nicéphore Mensah Glodjinon, Lamine Baba-Moussa, Farid Abdel Kader Baba-Moussa

**Affiliations:** 1 Food Science and Technology Training and Research Unit, Laboratory of Biotechnology and Food Microbiology, Department of Science and Technology, University Nangui Abrogoua, Abidjan, Côte d'Ivoire; 2 Laboratory of Biology and Molecular Typing in Microbiology, Department of Biochemistry and Cell Biology, Faculty of Sciences and Technology (FAST), University of Abomey-Calavi, Abomey-Calavi, Benin; 3 Laboratory of Microbiology and Food Technology, Department of Plant Biology, Faculty of Sciences and Technology (FAST), University of Abomey-Calavi, Abomey-Calavi, Benin; 4 Microbial Processes and Interactions, TERRA Teaching and Research Centre, BioEcoAgro, Gembloux Agro-Bio Tech, University of Liege, Gembloux, Belgium; 5 Faculty of Biotechnology, University of Agronomic Sciences and Veterinary Medicine of Bucharest, Bucharest, Romania

**Keywords:** antibacterial activity, Attiéké, climate change, *Enterococcus faecalis*, food biopreservation, hydrogen peroxide, lactic acid, lactic acid bacteria

## Abstract

**Background:**

Global climate change, particularly the rise in ambient temperatures, may promote the emergence and proliferation of pathogenic bacteria, thereby increasing food safety risks. Identifying robust bioprotective microorganisms that can retain their antimicrobial activity under elevated temperatures is therefore essential.

**Objective:**

This study investigated the biopreservation potential of lactic acid bacteria (LAB) isolated from *Attiéké* (cassava semolina) fermentation, focusing on their functional properties and antibacterial activity across a range of temperatures relevant to climate change scenarios.

**Methods:**

LAB isolates were evaluated for enzyme production, hemolytic activity, and the antibacterial activity of cell-free supernatants obtained after cultivation at 25 °C, 40 °C, 42 °C, 45 °C, and 50 °C. Additionally, their antibiotic susceptibility, lactic acid production, and hydrogen peroxide production were assessed. Selected strains were identified using polymerase chain reaction- restriction fragment length polymorphism (PCR-RFLP) followed by 16S rDNA sequencing.

**Results:**

Six LAB strains were identified, belonging to two species: *Lactobacillus plantarum* (LB 91, LB 92, LB 94, and LB 100) and *Enterococcus faecalis* (LB 95 and LB 152). All isolates were Gram-positive and exhibited γ-hemolysis. Strains LB 91, LB 92, LB 95, and LB 100 showed amylase and cellulase activities. The supernatant of strain LB 95 was the only one capable of inhibiting all tested pathogenic bacteria at all cultivation temperatures. All strains were sensitive to amikacin, minocycline, penicillin, erythromycin, and streptomycin. The highest lactic acid concentration (2.425 ± 0%) was recorded in LB 95 at 40 °C, while the highest hydrogen peroxide production (0.168 ± 0 mL) was observed in LB 94 at 25 °C.

**Conclusion:**

LB strains isolated from *Attiéké* demonstrated promising biopreservation properties at elevated temperatures. Among these strains, *Enterococcus faecalis* LB 95 exhibited the strongest antimicrobial activity and safety-associated traits, highlighting its potential as a bioprotective culture in a warming climate.

## 1 Introduction

Human activities and their associated climatic and environmental impacts are causing unprecedented rates of species decline, resulting in severe biodiversity loss (IPBES, 2019; Leclère et al., 2020; WWF, 2022) and threatening the stability of life-supporting systems on Earth (Ripple et al., 2023; IPCC, 2023). While the losses of species, communities, and habitats are well documented (United Nations Department of Economic and Social Affairs, 2018), microorganisms, despite their central role in ecosystem functioning, remain largely overlooked in discussions about climate change (Cavicchioli et al., 2019; Jansson and Hofmockel, 2020). Recent studies emphasize that microbial responses to climate change may have profound consequences for ecosystem stability, biogeochemical cycles, and human health (Anuoluwa et al., 2024).

Climate change is characterized by extreme events such as torrential rainfall, droughts, and rising temperatures, all of which have serious consequences for human, animal, and plant health, as well as for environmental balance (IPCC, 2021). Increasing temperatures, in particular, may promote the emergence and proliferation of pathogenic bacteria, thereby posing significant food safety risks.

Several foodborne pathogens, including Campylobacter, Salmonella, *Listeria monocytogenes*, *Clostridium perfringens*, and *Vibrio* spp., have been linked to climate variability (von der Pahlen and Mukherjee, 2019; Dubois-Brissonnet and Guillier, 2020).

For example, the incidence of salmonellosis increases by approximately 12% for every degree that temperatures exceed an average weekly or monthly level of 6 °C (Stephen and Barnett, 2016). Rising ocean temperatures also increase the density of *Vibrio* spp., including species responsible for cholera, which in turn leads to higher rates of seafood-associated infections (von der Pahlen and Mukherjee, 2019). Recent climate-driven outbreaks of *Vibrio vulnificus* and *Vibrio parahaemolyticus* in Europe and North America further illustrate this ongoing trend (Jayakumar et al., 2024). Sudden decreases in salinity caused by intense rainfall can also trigger sharp increases in human-pathogenic vibrios in coastal ecosystems (Dubois-Brissonnet and Guillier, 2020).

Foodborne pathogens are therefore particularly sensitive to changes in climate (Lake, 2017; Cavicchioli et al., 2019). At the same time, the effectiveness of antibiotics is becoming increasingly limited. Climate change is predicted to accelerate the development of antibiotic resistance in several human pathogens (MacFadden et al., 2018; McGough et al., 2020). Recent global analyses confirm that higher ambient temperatures are associated with increased resistance in *E. coli*, *Klebsiella pneumoniae*, and *Staphylococcus aureus* (McGough et al., 2020). Proposed mechanisms for this phenomenon include temperature-enhanced horizontal gene transfer, increased pathogen growth rates, and greater persistence of pathogens in the environment (MacFadden et al., 2018). Given these challenges, the use of biological tools such as beneficial microorganisms offers an ecological and sustainable strategy. Lactic acid bacteria (LAB) are of particular interest due to their central role in food fermentation and their well-documented health benefits (Vinderola et al., 2022). Traditional fermented foods such as Attiéké—a cassava-based product from Côte d’Ivoire—are rich sources of LAB with potential probiotic and bioprotective properties (N’Dri et al., 2023). Attiéké is a staple food widely consumed by several ethnic groups in southern Côte d’Ivoire (Mobio et al., 2023). Although traditionally Ivorian, its production and consumption now extend nationwide and increasingly reach international markets (Djeni et al., 2015). The product is obtained through a complex artisanal process in which fermentation plays a central role. This fermentation is initiated by a natural starter known as “Mangnan,” derived from the spontaneous fermentation of cassava roots (Egbune et al., 2023). Despite being uncontrolled, this fermentation is essential for the sensory characteristics of Attiéké (Toka et al., 2018) and supports the growth of diverse LAB populations (Mokoena et al., 2021; Halake and Chinthapalli, 2020). Given the increasing emergence of foodborne pathogens in the context of climate change, this study investigated the antibacterial activity of LAB isolated from Attiéké against emerging pathogens, with a particular focus on their performance at elevated cultivation temperatures.

### 1.1 Bacterial strains and culture conditions

Six lactic acid bacteria (LAB) strains were selected from an initial collection of 100 isolates obtained from the traditional Attiéké ferment (“Mangnan”) collected in Cocody-Blockhauss (Abidjan, Côte d’Ivoire). The isolation process was performed on de Man, Rogosa, and Sharpe (MRS) agar, followed by incubation at 37 °C for 24 h under anaerobic conditions.

### 1.2 Phenotypic characterization

#### 1.2.1 Microscopy and enzymatic activities

Gram staining was performed using a commercial kit (crystal violet, Lugol’s iodine, alcohol, fuchsin). Cell morphology was examined under a light microscope at 100X magnification (oil immersion) (Harley, 2017). Oxidase activity was assessed using N, N-dimethyl-p-phenylenediamine reagent, and catalase activity was evaluated by adding hydrogen peroxide (MacFaddin, 2000). Enzymatic activities were assessed on selective media. Amylase activity was evaluated on MRS agar supplemented with 1% soluble starch, and clear halos after iodine staining indicated starch hydrolysis Xiao et al. (2006). Cellulase activity was determined on MRS agar containing 1% carboxymethylcellulose; plates were stained with 0.1% Congo red and washed with 1 M NaCl, and yellow–orange halos were interpreted as positive cellulase activity (Kasana et al., 2008). Lipase activity was assessed on MRS agar containing Tween 80 (0.25 mL), CaCl₂·H₂O (0.01%), and rhodamine (0.0001%), with pink–orange fluorescence under UV light (350 nm) indicating lipase production (Kouker and Jaeger, 1987). Protease activity was evaluated on 1% skim milk agar, where clear zones reflected casein hydrolysis (Harrigan, 1998).

### 1.3 Hemolytic activity

The hemolytic activity of the LAB isolates was determined using the procedure described by Yadav et al. (2016). Hemolysis was assessed by spotting 5 μL of each LAB culture onto Columbia agar supplemented with 5% (w/v) sheep blood (Oxoid, UK). Plates were incubated at 37 °C for 48 h. Hemolysis was classified as β- (clear halo), α- (green halo), or γ-hemolysis (no halo).

### 1.4 Preparation of cell-free supernatants

LAB strains were cultured in MRS broth at 25 °C, 40 °C, 42 °C, 45 °C, and 50 °C for 48 h. Cultures were centrifuged at 10,000 × g for 5 min at 4 °C, and the resulting supernatants were filtered through sterile 0.22 μm membranes to obtain cell-free supernatants (CFSs) (Coulibaly et al., 2023).

### 1.5 Antibacterial activity assay

Antagonistic activity was evaluated against *Staphylococcus aureus* ATCC 29213, *Escherichia coli* ATCC 15922, *Listeria monocytogenes*, *Pseudomonas aeruginosa*, and *Bacillus subtilis* (Merck, Germany) by the agar well diffusion method described by Balouiri et al. (2016). Pathogen suspensions (OD₆₀₀ = 0.2 ± 0.05; ~10^7^–10^8^ CFU/mL) were mixed with molten tryptic soy agar (TSA, 45 °C) and poured into Petri dishes. Wells (6 mm) were filled with 100 μL of CFS. Plates were incubated at 37 °C for 24 h. Inhibition zones ≥1 mm were considered positive.

### 1.6 Titratable acidity

LAB strains were cultured at 25 °C, 40 °C, and 42 °C for 48 h. CFS was titrated with 0.1 N NaOH, and lactic acid content was expressed as a percentage (%) (AOAC International, 1995). Measurements were performed in triplicate.

### 1.7 Quantification of hydrogen peroxide

Hydrogen peroxide concentration in CFS was determined by titration with 0.1 N potassium permanganate (KMnO₄) using the method described by Huckaba and Keyes, (1948). Results were expressed in milliliters (mL). Analyses were performed in triplicate.

### 1.8 Antibiotic susceptibility testing

Antibiotic susceptibility was assessed using the disc diffusion method following CLSI guidelines (CLSI, 2016). The following antibiotics were tested: β-lactams: penicillin (6 μg) and cefoxitin (30 μg); aminoglycosides: gentamicin (15 μg), amikacin (30 μg), and streptomycin (500 μg); quinolones: ciprofloxacin (5 μg) and levofloxacin (5 μg); cyclines: minocycline (30 μg); cephalosporins: cefuroxime sodium (30 μg); aminopenicillins: amoxicillin/clavulanic acid (20/10 μg); macrolides: erythromycin (15 μg); polymyxins: colistin (50 μg); and glycopeptides: vancomycin (5 μg). Fresh LAB cultures (OD₆₀₀ = 0.2 ± 0.05) were spread on MRS agar. Plates were incubated at 37 °C for 48 h. Inhibition zones were interpreted as sensitive (S), intermediate (I), or resistant (R) according to the CLSI criteria.

### 1.9 Molecular identification of LAB strains

LAB strains were grown in MRS broth for 48 h at 37 °C. Cells were harvested by centrifugation (5,000 × g, 10 min). Genomic DNA was extracted using the ZR Fungal/Bacterial DNA Kit (Zymo Research, United States). The 16S rRNA gene was amplified using primers: 27F: 5′-AGAGTTTGATCCTGGCTCAG-3′ and 1492R: 5′-GGTACCTTGTTACGACTT-3′. PCR products were sequenced bidirectionally (Eurofins, Germany). Sequences were compared with NCBI GenBank entries using BLAST. Phylogenetic analysis was performed using the neighbor-joining method (Saitou and Nei, 1987) in MEGA X (Kumar et al., 2018).

### 1.10 Experimental factorial design

The experiments were conducted using a factorial design in which LAB strain and incubation temperature were treated as fixed factors, and the measured technological or antimicrobial traits were response variables. Six LAB strains (LB 91, LB 92, LB 94, LB 95, LB 100, LB 152) and three incubation temperatures (25 °C, 40 °C, 42 °C) were combined in a 6 × 3 factorial scheme for lactic acid and hydrogen peroxide production, yielding 18 treatment combinations, each tested in duplicate with three analytical replicates per treatment. For antibacterial activity, a 6 × 3 × 5 factorial structure was applied, combining six LAB strains, three incubation temperatures (25 °C, 40 °C, 42 °C), and five indicator pathogens (*Staphylococcus aureus* ATCC 29213, *Escherichia coli* ATCC 15922, *Listeria monocytogenes* ATCC 19114, *Pseudomonas aeruginosa* ATCC 27853, *Bacillus subtilis*). In this case, the inhibition zone diameter was used as the response variable, and pathogen effect was analyzed within each strain–temperature combination. For antibiotic susceptibility, all 6 strains were tested against 13 antibiotics in a 6 × 13 factorial arrangement, and inhibition zone diameter was recorded as the response. This design allowed simultaneous evaluation of main effects (strain, temperature, pathogen, or antibiotic) and their interactions, which were subsequently analyzed by two-way ANOVA as described below.

### 1.11 Statistical analysis

All experiments were performed in duplicate, with each measurement conducted in triplicate. Data are expressed as mean ± standard deviation (SD). Statistical significance (*p* < 0.05) was determined using two-way ANOVA followed by Tukey’s *post-hoc* test. The clustered heatmap with associated dendrograms was generated using the R statistical environment (version 4.x; R Core Team, Vienna, Austria). Data preprocessing, scaling, clustering, and visualization were performed using functions from the stats, pheatmap, and ComplexHeatmap packages.

## 2 Results

### 2.1 Characteristics of LAB isolates

**Figure 1:**
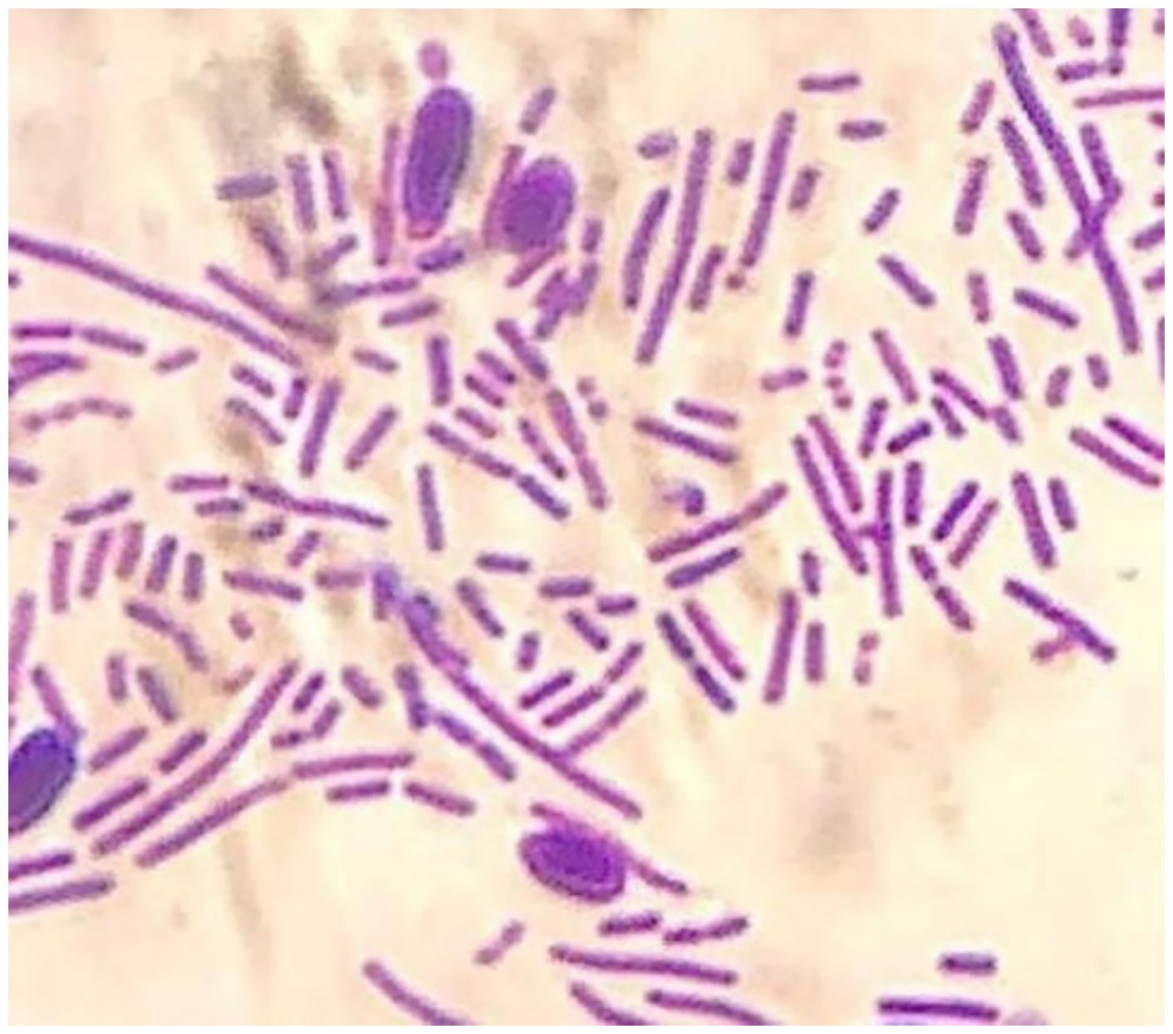
Gram-stained morphology of the bacterial LB 95 isolate.

All LAB isolates were Gram-positive bacilli and tested negative for catalase, oxidase, protease, and lipase activities (Figure 1). This micrograph showed the cellular morphology of the isolate after Gram staining. The preparation was observed under bright-field microscopy at high magnification. Cells appear as purple rod-shaped bacilli, indicating a Gram-positive reaction due to retention of crystal violet by a thick peptidoglycan cell wall. The figure illustrates cell shape, arrangement, and staining behavior, which are key variables used to confirm culture purity and support taxonomic identification. LB 91, LB 92, LB 95, and LB 100 isolates showed positive amylase and cellulase activities, whereas LB 94 and LB 152 did not exhibit these enzymatic functions (Table 1).

**Table 1:** Enzymatic profile of LB strains.

Strains	Catalase	Oxydase	Amylase	Lipase	Protease	Cellulase
LB 91	−	−	+	−	−	+
LB 92	−	−	+	−	−	+
LB 94	−	−	−	−	−	−
LB 95	−	−	+	−	−	+
LB 100	−	−	+	−	−	+
LB 153	−	−	−	−	−	−

(+): Enzyme production; (−): absence of production.

### 2.2 Hemolytic activity

Hemolysin is an enzyme that lyses red blood cells and may cause adverse effects, such as anemia, antigen–antibody reactions, and other health disorders (Thao et al., 2021). In this study, all LAB isolates exhibited γ-hemolysis, characterized by the absence of clear or green halos around colonies on blood agar. This non-hemolytic profile indicates that the isolates are safe for potential use as biopreservative cultures.

### 2.3 Antibacterial activity of LAB isolates at elevated temperatures

**Figure 2:**
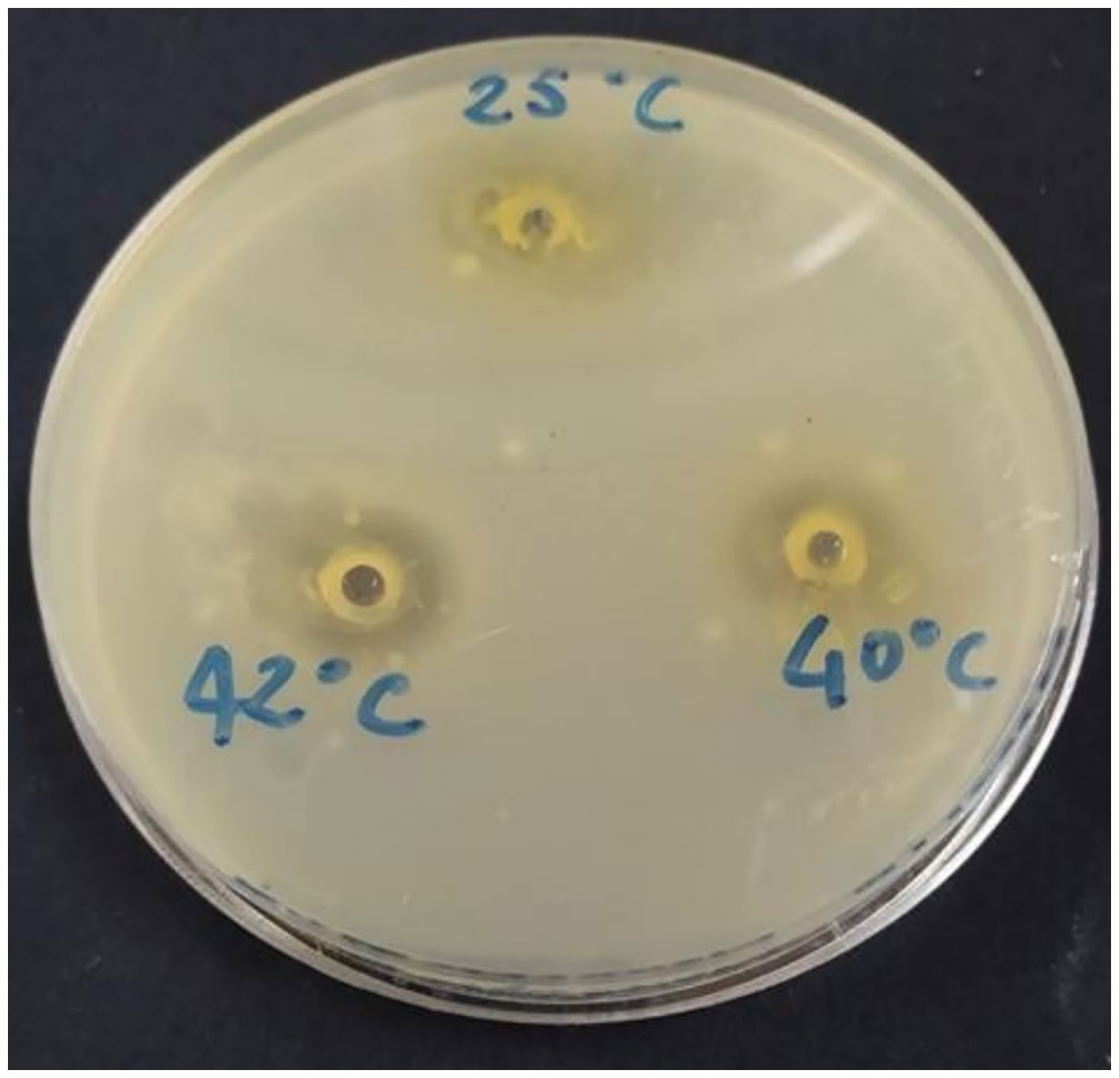
Inhibition of *Listeria monocytogenes* ATCC19114 by the supernatant of LB 95 strain produced at different temperatures (25 °C, 40 °C, and 42 °C).

Table 2 presents the inhibition zone diameters (mm) produced by six lactic acid bacteria (LAB) isolates (LB 91, LB 92, LB 94, LB 95, LB 100, and LB 152) against five reference pathogenic strains at three incubation temperatures (25 °C, 40 °C, and 42 °C). Values are expressed as mean ± standard deviation based on triplicate measurements. Different lowercase superscripts indicate statistically significant differences between treatments according to Tukey’s test (*p* < 0.05). For all pathogens and temperatures, the ANOVA results (*Pr > F* = 0.000) confirm that the effects of the tested factors were statistically significant. Across all pathogens, antimicrobial activity varied depending on the LAB isolate and incubation temperature. Several isolates, including LB 91, LB 100, and LB 152, showed no inhibition under certain conditions, particularly at 25 °C (LB 91: 0 ± 0 mm; LB 100: 0 ± 0 mm; LB 152: 0 ± 0 mm against *B. subtilis*). In contrast, LB 92, LB 94, and LB 95 isolates produced measurable inhibition zones across multiple pathogens and temperatures. LB 92 isolate inhibited *B. subtilis* with diameters of 14.6 ± 0.5 mm at 25 °C and 14.3 ± 0.5 mm at 40 °C, while BL 94 isolate produced inhibition zones of 11 ± 1 mm at 25 °C and 13 ± 1 mm at 40 °C. LB 95 isolate consistently exhibited the largest inhibition zones, notably against *Pseudomonas aeruginosa* (11 ± 1 mm at 25 °C; 9.3 ± 0.5 mm at 40 °C) and *Listeria monocytogenes* (10 ± 1 mm at 25 °C; 9.6 ± 1.1 mm at 40 °C). For most isolates, inhibition was higher at 40 °C than at 25 °C and 42 °C. Differences in susceptibility were also observed among the pathogenic strains. *Bacillus subtilis* and *Listeria monocytogenes* generally showed larger inhibition zones, such as 14.6 ± 0.5 mm (LB 92 25 °C) and 10 ± 1 mm (LB 95 25 °C), whereas *Escherichia coli* and *Pseudomonas aeruginosa* displayed lower sensitivity, with several values ranging between 3 and 6 mm depending on the isolate and temperature. Overall, the data indicated that antimicrobial activity depended significantly on the LAB isolate, the incubation temperature, and the target pathogen. Notably, LB 95 isolate inhibited all indicator pathogens (Table 2). To complement these quantitative results, a clustered heatmap was generated to visualize global activity patterns across strains and temperatures (Figure 2). The clustered heatmap revealed distinct patterns of antibacterial activity among the LB strains tested at different incubation temperatures (25 °C, 40 °C, 42 °C) (Figure 2). The color gradient, ranging from blue (low standardized activity) to red (high activity), highlighted clear strain- and temperature-dependent variations. Strains LB95 and LB152 exhibited the highest overall antibacterial potential, particularly at 40 °C, where strong inhibition was observed against *Bacillus subtilis* and *Staphylococcus aureus*. In contrast, LB94 and LB92 showed moderate activity, with reduced inhibition at 25 °C and 42 °C. The clustering dendrogram grouped the strain–temperature combinations into two major clusters: one comprising highly active profiles (LB 95 40 °C, LB 152 40 °C, LB 100 42 °C) and another including low-activity conditions (LB 94 25 °C, LB 92 25 °C). Among the target bacteria, *B. subtilis* and *S. aureus* were the most sensitive, while *Pseudomonas aeruginosa* and *Escherichia coli* exhibited lower susceptibility. The hierarchical clustering thus confirmed that both strain identity and incubation temperature significantly influenced the antibacterial performance, consistent with the quantitative inhibition patterns observed in previous assays.

**Table 2:** Antibacterial activity of LAB strains, expressed as inhibition zone diameters (mm), against five indicator bacteria at 25 °C, 40 °C, and 42 °C.

Strains	*B. subtilis* (Merck)			*E. coli* ATCC15922			*P. aeruginosa* ATCC27853			*L. monocytogenes* ATCC19114			*S. aureus* ATCC29213		
	25 °C	40 °C	42 °C	25 °C	40 °C	42 °C	25 °C	40 °C	42 °C	25 °C	40 °C	42 °C	25 °C	40 °C	42 °C
LB 91	0 ± 0a	0 ± 0a	0 ± 0a	7.6 ± 0.5a	9.3 ± 0.5a	3.6 ± 0.5a	4 ± 0a	6 ± 1a	1.3 ± 0.5a	0 ± 0a	0 ± 0a	0 ± 0a	0 ± 0a	0 ± 0a	0 ± 0a
LB 92	14.6 ± 0.5b	14.3 ± 0.5b	12.3 ± 0.5b	6.3 ± 0.5ab	4.6 ± 0.5b	4.3 ± 0.5ab	3.33 ± 0.5a	3.3 ± 0.5ab	2.3 ± 0.5a	4.3 ± 0.5b	3.6 ± 0.5b	4.3 ± 0.5b	0 ± 0a	0 ± 0a	0 ± 0a
LB 94	11 ± 1c	13 ± 1b	12.66 ± 0.57b	4.6 ± 0.5c	4.3 ± 0.5b	6. ± 0.5b	4.6 ± 0.5a	4.6 ± 0.57a	4.6 ± 0.5b	4.6 ± 0.5b	4.6 ± 0.5b	4.6 ± 0.5b	0 ± 0a	4.66 ± b	0 ± 0a
LB 95	13 ± 1bc	6.33 ± 0.57c	6.66 ± 0.57c	4.3 ± 0.5c	5 ± 1 b	5 ± 1ab	11 ± 1b	9.3 ± 0.5c	7.33 ± 0.c	10 ± 1c	9.6 ± 1.1c	10.3 ± 1.2c	9 ± 1b	9 ± 0c	9.3 ± 0.5b
LB 100	0 ± 0 a	0 ± 0 a	0 ± 0a	9.6 ± 0.5d	10 ± 1a	9.66 ± 1c	8.3 ± 0.5c	5 ± 1b	4.6 ± 0.5b	0 ± 0d	0 ± 0d	0 ± 0d	0 ± 0a	0 ± 0a	0 ± 0a
LB 153	0 ± 0 a	0 ± 0 a	0 ± 0a	4.33 ± 1.15c	4 ± 1 b	4.33 ± 0.57ab	3 ± 0.5d	0 ± 0d	0 ± 0d	0 ± 0d	0 ± 0d	0 ± 0d	0 ± 0a	0 ± 0a	0 ± 0a
Pr > F	0.000	0.000	0.000	0.000	0.000	0.000	0.000	0.000	0.000	0.000	0.000	0.000	0.000	0.000	0.000
Significant	Yes	Yes	Yes	Yes	Yes	Yes	Yes	Yes	Yes	Yes	Yes	Yes	Yes	Yes	Yes

Values expressed as mean ± standard deviation of triplicate measurements. In the same column, different lowercase superscripts indicate significant differences (*P* < 0.05, Tukey’s test).

### 2.4 Antibiotic susceptibility of LAB isolates

**Figure 3:**
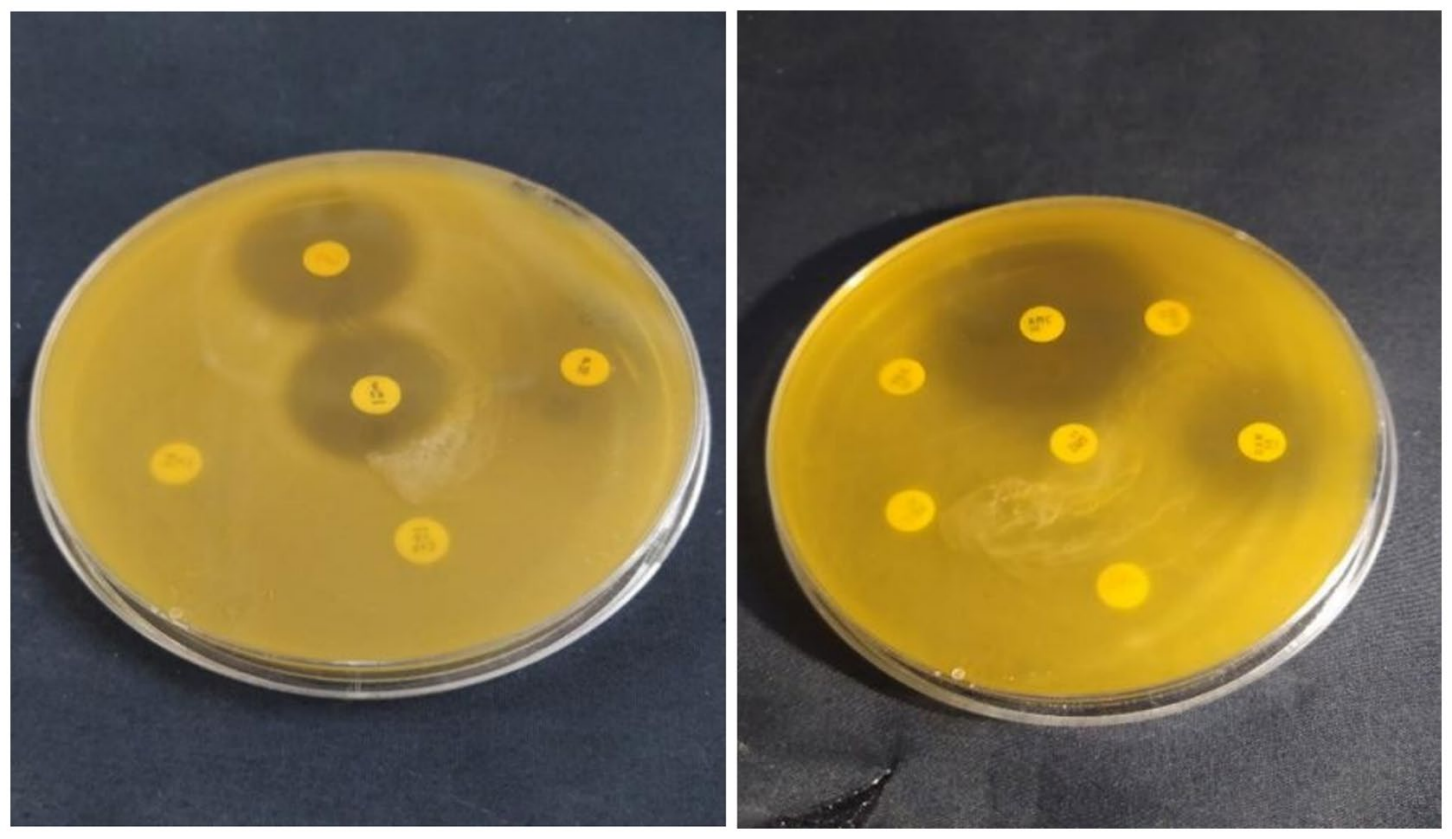
Antibiotic susceptibility of the LB 95isolate assessed by disk diffusion.

Antibiotic susceptibility was evaluated using the disk diffusion method with 13 antibiotics representing 10 antibiotic classes (Figure 3). This figure shows the inhibition zones produced by antibiotic disks placed on an agar plate inoculated with the bacterial isolate. Each disk corresponds to a specific antibiotic and concentration, as indicated by the printed labels. Clear halos surrounding the disks represent growth inhibition and susceptibility, while the absence of a halo reflects resistance.

**Figure 4:**
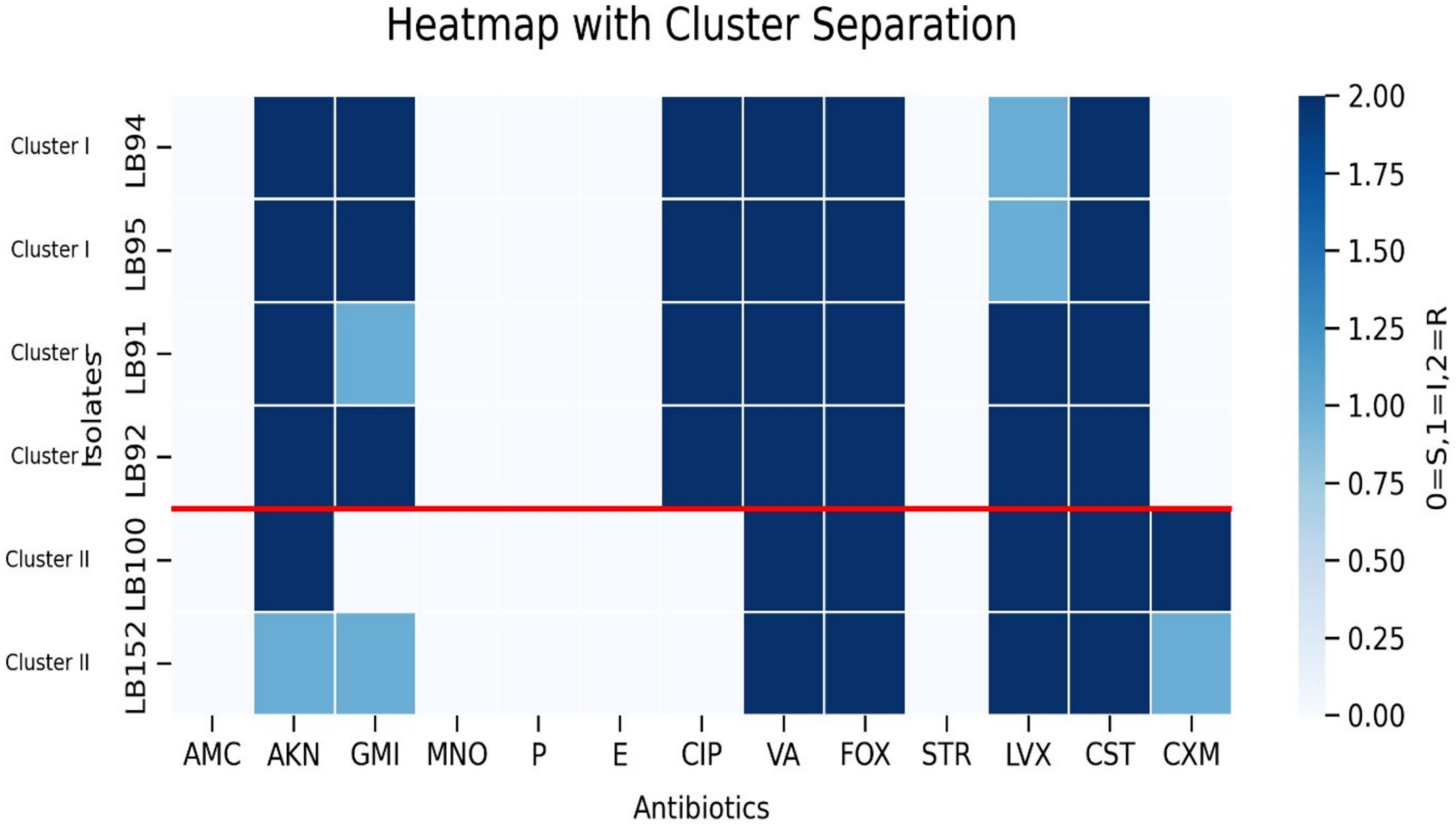
Heatmap representing the antibiotic susceptibility patterns of six lactic acid bacteria (LAB) isolates (LB 91, LB 92, LB 94, LB 95, LB 100, and LB 152) against 13 antibiotics. Inhibition diameters were interpreted according to standard criteria and classified as sensitive (S), intermediate (I), or resistant (R), and subsequently encoded as 0, 1, and 2, respectively. The color gradient ranges from light blue (sensitive) to dark blue (resistant).

The variables represented include the type of antibiotic, its concentration, and the diameter of the inhibition zone. Figure 4 shows the hierarchical clustering of antibiotic susceptibility profiles, which revealed two distinct strain clusters and three antibiotic response groups, confirming the strain × antibiotic interaction observed in the ANOVA (Table 3). Row dendrogram (strains): hierarchical clustering (Ward method) was applied to group isolates based on similarity in their resistance profiles. The dendrogram was cut to define two main clusters, which are indicated on the heatmap and separated by a horizontal line: Cluster I (LB 91, LB 92, LB 94, LB 95), characterized by higher resistance to selected antibiotics such as ciprofloxacin and aminoglycosides, and Cluster II (LB 100, LB 152), showing relatively more variable responses and increased susceptibility to certain antibiotics. Overall, all isolates exhibited consistent sensitivity to β-lactams and macrolides, while resistance was uniformly observed for vancomycin, cefoxitin, and colistin. The turquoise-to-blue gradient clearly visualizes susceptibility intensity, highlighting the differential resistance patterns among strains and confirming the significant strain × antibiotic interaction detected in the factorial analysis.

**Table 3:** Summary of two-way ANOVA on antibiotic susceptibility scores (1 = resistant, 2 = intermediate, and 3 = sensitive) of lactic acid bacteria isolates.

Source of variation	Factor levels	Effect on susceptibility score	Significance	Interpretation
Strain	6 LAB strains (LB 91, LB 92, LB 94, LB 95, LB 100, LB 152)	Main effect	*P* < 0.05	Susceptibility differs significantly between strains
Antibiotic	13 antibiotics (10 classes)	Main effect	*P* < 0.01	Strong differences in susceptibility between antibiotics
Strain × antibiotic	6 × 13 combinations	Interaction	*P* < 0.01	Strain response depends on the specific antibiotic tested

Data were analyzed using a two-way factorial ANOVA with strain and antibiotic as fixed factors. Susceptibility categories (sensitive, intermediate, resistant) were coded as 3, 2, and 1, respectively. Significant main effects and interactions indicate that both the type of antibiotic and the identity of the strain, as well as their combination, influence the susceptibility profile.

### 2.5 Titratable acidity and hydrogen peroxide production

**Figure 5:**
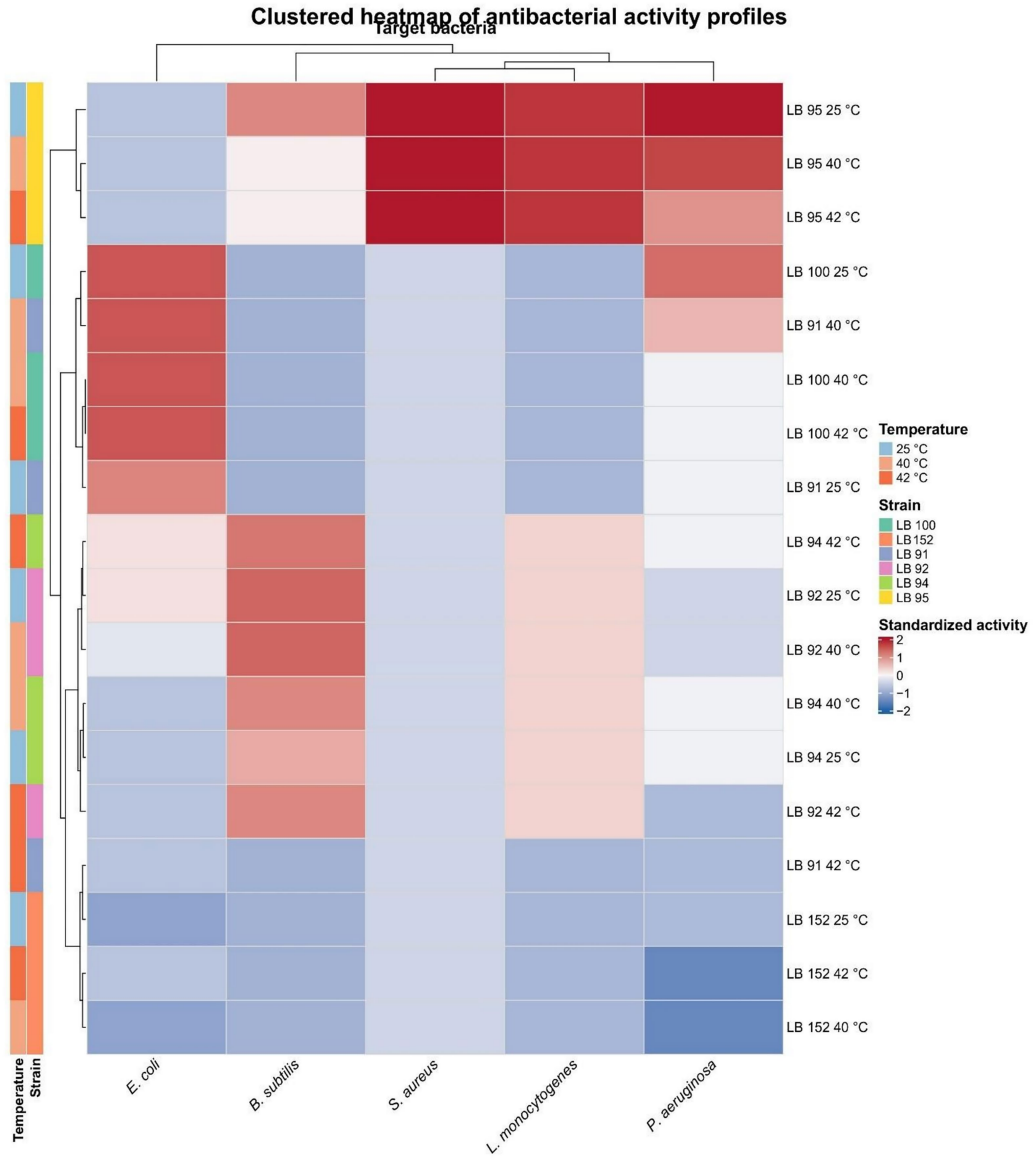
Clustered heatmap of antibacterial activity profiles of *Lactiplantibacillus* strains at different incubation temperatures (25 °C, 40 °C, 42 °C) against five target bacteria (*E. coli*, *B. subtilis*, *S. aureus*, *L. monocytogenes*, and *P. aeruginosa*).

Figure 5 shows the titratable acidity rate, expressed in lactic acid (%) and hydrogen peroxide (mL), by six LAB strains (LB 91, LB 92, LB 94, LB 95, LB 100, LB 152) incubated at three temperatures (25 °C, 40 °C, 42 °C). Patterned bars represent lactic acid production, while solid bars represent hydrogen peroxide levels. This figure highlights strain-dependent and temperature-dependent variations in metabolic activity. LAB strains selected for their antibacterial activity produced antimicrobial metabolites, including titratable acidity (% lactic acid) and hydrogen peroxide. Titratable acidity (% lactic acid) was produced in higher quantities than hydrogen peroxide. Production patterns varied among strains and temperatures, with no consistent temperature-dependent trend. Titratable acidity (%) and hydrogen peroxide (mL) production were analyzed using a two-way ANOVA with six strains and temperature (25 °C, 40 °C, 42 °C) as fixed factors. Each condition included triplicate measurements. Significant main effects were detected for strain (*p* < 0.001) and temperature (*p* < 0.01), as well as a significant strain × temperature interaction (*p* < 0.001), indicating that the metabolic response to temperature varied among isolates. Tukey’s post-hoc test was used to compare means, and different lowercase letters above bars denote statistically significant differences (*p* < 0.05). The highest titratable acidity value was recorded for strain LB 95 at 40 °C (2.42 ± 0%), significantly exceeding all other strains (*p* < 0.05), whereas the lowest was observed for strain LB 152 (0.53 ± 0%).

Hydrogen peroxide levels were lower overall but showed pronounced strain differences. LB 94 produced the highest H₂O₂ at 25 °C (0.168 ± 0 mL) and 42 °C (0.165 ± 0 mL), significantly higher than other strains (*p* < 0.05).

### 2.6 Identification of LAB isolates

**Figure 6:**
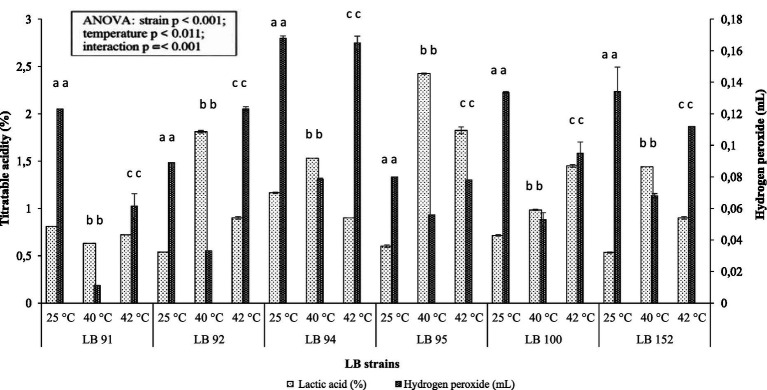
Titratable acidity and hydrogen peroxide production by LAB strains at different temperatures. Values are expressed as means ± standard deviation of three independent measurements (*n* = 3). Different letters above the bars indicate statistically significant differences among conditions (*p* < 0.05).

**Figure 7:**
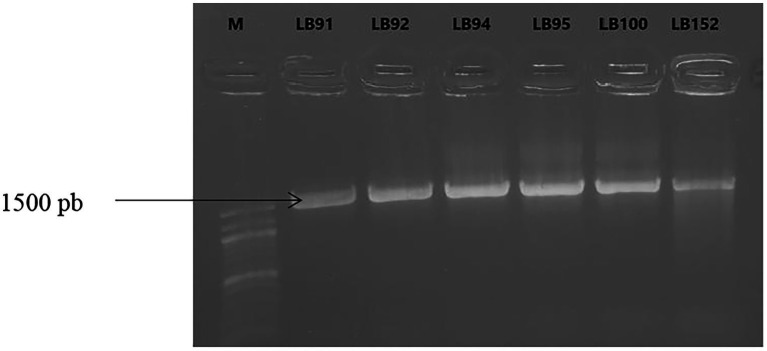
PCR amplification of the 16S rDNA region of selected LB strains.

**Figure 8:**
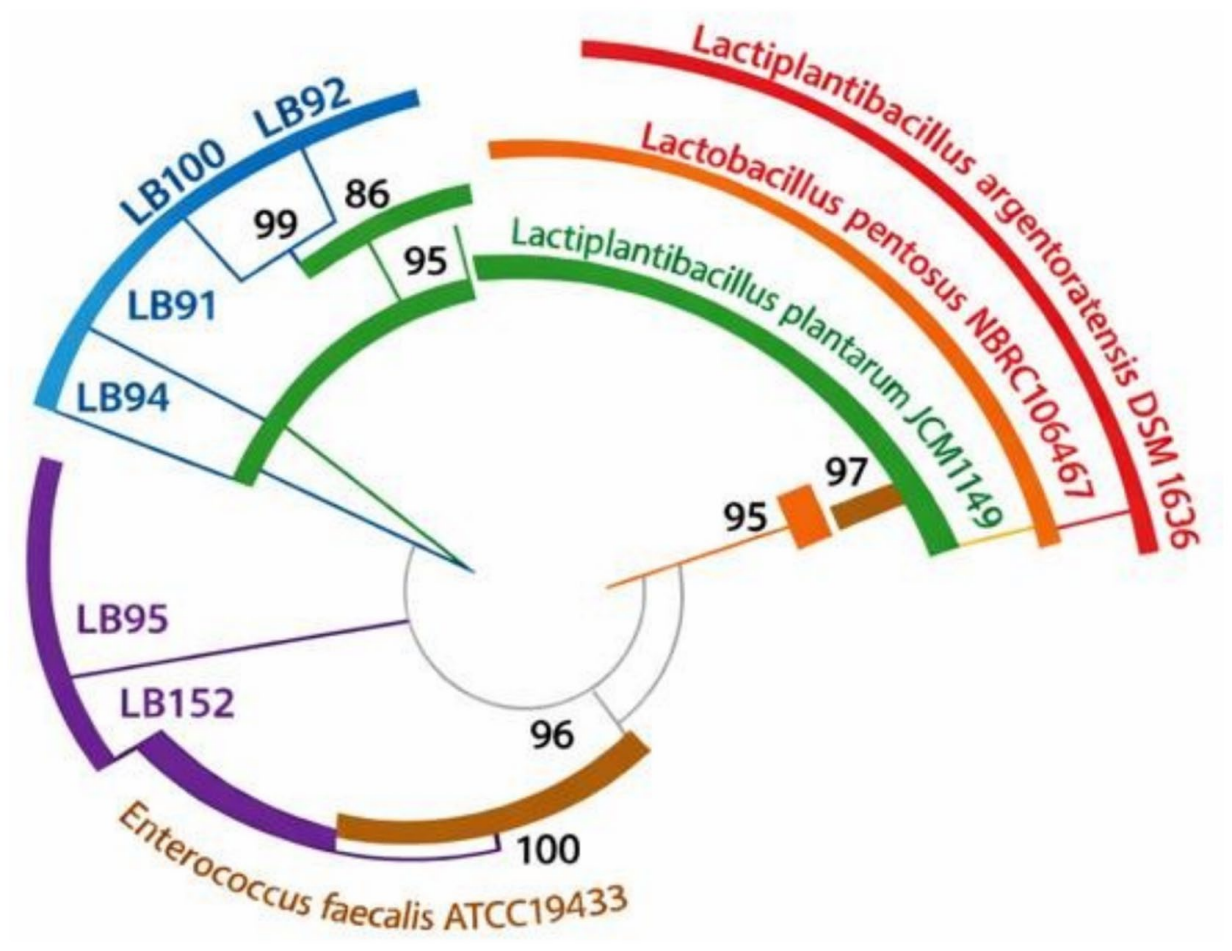
Circular phylogenetic tree shows the evolutionary relationships between the LAB isolates (LB 91, LB 92, LB 94, LB 95, LB 100, LB 152) and reference strains obtained from GenBank. The tree was constructed using 16S rDNA sequences and a neighbor-joining algorithm with bootstrap analysis to assess branch reliability.

This figure shows the PCR amplification profiles of the 16S rDNA region obtained from selected LAB isolates (LB 91, LB 92, LB 94, LB 95, LB 100, LB 152). DNA fragments were separated by agarose gel electrophoresis alongside a molecular weight ladder used as a size reference. Each lane corresponds to one isolate, and the presence of a single clear band of the expected size (~1.5 kb) confirms successful amplification of the 16S rDNA region. Band intensity reflects amplification efficiency, while lane labels identify the strains analyzed (Figure 6). BLAST analysis of the partial sequences revealed high similarity values, and all sequences were deposited in the NCBI GenBank database (accession numbers PZ062416–PZ062421) (Table 4). Strains LB 91, LB 92, LB 94, and LB 100 were identified as *Lactiplantibacillus plantarum*, with sequence similarity ranging from 99.45 to 100%. Strains LB 95 and LB 152 were identified as *Enterococcus faecalis*, with similarity values between 99.34 and 99.92%. Phylogenetic analysis (Figure 7) confirmed the presence of two main species clusters: *Lactiplantibacillus plantarum* (LB 91, LB 92, LB 94, LB 100) (blue), closely related to *L. plantarum* JCM 1149, as confirmed by bootstrap values ranging from 86 to 100%. *Enterococcus faecalis* (LB 95, LB 152) (purple), clustering with *E. faecalis* ATCC 19433, strongly supported by 100% bootstrap value (Figure 8).

**Table 4:** LB species and accession numbers.

Strains	Species	Accession numbers
LB 91	*Lactobacillus plantarum*	PZ062416
LB 92	*Lactobacillus plantarum*	PZ062417
LB 94	*Lactobacillus plantarum*	PZ062418
LB 95	*Enterococcus faecalis*	PZ062419
LB 100	*Lactobacillus plantarum*	PZ062420
LB 152	*Enterococcus faecalis*	PZ062421

## 3 Discussion

Lactic acid bacteria (LAB) are widely recognized for their safety, metabolic versatility, and strong antibacterial properties, making them valuable natural alternatives to chemical preservatives in food systems (Rajanikar et al., 2021). In the context of climate change, where rising temperatures may favor the emergence and persistence of foodborne pathogens, the ability of LAB to maintain growth and antimicrobial activity under thermal stress becomes particularly relevant. In this study, LAB isolates obtained from Attiéké ferment retained antibacterial activity at elevated temperatures, highlighting their potential as biopreservative agents in warming environments. The six isolates were identified as *Lactiplantibacillus plantarum* (LB 91, LB 92, LB 94, LB 100) and *Enterococcus faecalis* (LB 95, LB 152), consistent with previous reports describing the dominance of *L. plantarum* and *Enterococcus* spp. in cassava fermentations and other African fermented foods (Atuna et al., 2025; Adesulu-Dahunsi et al., 2022). The functional properties observed in our isolates aligned with earlier studies. *L. plantarum* O2 produces thermostable antimicrobial metabolites active between 40 and 120 °C (Huang et al., 2023), while *L. plantarum* O1 grows at 45 °C and inhibits a broad spectrum of pathogens (Čanak et al., 2018). Non-virulent *E. faecalis* strains have also been shown to be safe and effective in food systems (John, 2026). Enzymatic profiling revealed that LB 91, LB 92, LB 95, and LB 100 exhibited both amylase and cellulase activities, whereas LB 94 and LB 152 lacked these functions. Carbohydrate-degrading enzymes enhance LAB competitiveness and metabolic activity in plant-based fermentations, particularly under environmental stress (Derunets et al., 2024). Although enzyme production does not directly determine antimicrobial activity, the ability to hydrolyze complex substrates may support sustained metabolite synthesis under thermal variation. Increasing temperatures are expected to influence microbial dynamics in fermented foods and may favor the growth of heat-tolerant pathogens. LAB strains capable of maintaining metabolic activity and antimicrobial production at elevated temperatures are therefore relevant for biopreservation strategies. Several isolates in this study, particularly LB 92 and LB 95, retained inhibitory activity at 40 °C and 42 °C, consistent with recent findings showing that certain LAB maintain acidification and bacteriocin production under heat stress (He et al., 2025). The combined enzymatic and antimicrobial profiles suggested that LB 92, LB 95, and LB 100 possess functional traits suitable for biopreservation applications in warming food environments. The consistent clustering of LB 95 across all temperature conditions highlighted its superior inhibitory performance compared with the other isolates, indicating a robust capacity to produce antimicrobial metabolites under varying environmental conditions. Recent studies published between 2023 and 2025 further support these observations. Rocchetti et al. (2023) demonstrated that *L. plantarum* and *Enterococcus* spp. activate heat shock protein pathways that preserve metabolic activity under thermal fluctuations, consistent with the ability of our isolates to maintain antibacterial activity at 40–42 °C. Turgis et al. (2016) reported that *P. acidilactici* MM33 produced the highest bacteriocin yield at 45 °C. Reviews have also highlighted the diversity and thermostability of LAB antimicrobial metabolites, mainly bacteriocins (Wei et al., 2025; Karbowiak et al., 2025). The antibacterial profiles of LB 91, LB 92, LB 94, and LB 100 are consistent with findings on *L. plantarum* strains isolated from African fermented foods, which frequently harbor plantaricin operons and exhibit broad inhibitory spectra (Oguntoyinbo and Narbad, 2015). Phylogenetic analysis revealed two well-defined clusters corresponding to *L. plantarum* (LB 91, BL 92, LB 94, LB 100) and *E. faecalis* (LB 95, LB 152). The *L. plantarum* isolates grouped tightly with the reference strain JCM 1149, with similarity values ranging from 99.45 to 100%, confirming the robustness of their taxonomic assignment. LB 91 and LB 92 showed particularly close proximity, suggesting a recent common ancestor or similar ecological adaptation. LB 95 and LB 152 clustered with *E. faecalis* ATCC 19433, a non-virulent reference strain commonly associated with food fermentations rather than clinical settings. Metabolite quantification revealed significant effects of strain and temperature on lactic acid and hydrogen peroxide production, with a strong strain × temperature interaction. Lactic acid concentrations were consistently higher than hydrogen peroxide levels, confirming acidification as the dominant antimicrobial mechanism. LB 95 produced the highest lactic acid concentration at 40 °C, consistent with its broad and stable antibacterial profile. LB 152 exhibited the lowest lactic acid levels, reflecting limited fermentative capacity. Hydrogen peroxide production followed a distinct pattern: LB 94 generated the highest levels at both 25 °C and 42 °C, demonstrating a robust oxidase-mediated response across a wide thermal range. The ability of LB 95 and LB 94 to maintain or enhance antimicrobial metabolite production at 40–42 °C supports their potential use as climate-resilient biopreservative cultures. Dias et al. (2025) reported that bovicin HC5 production in *Streptococcus equinus* HC5 was enhanced when *Streptococcus equinus* HC5 was cultivated at 42 °C in adaptive laboratory evolution under thermal stress. The antimicrobial activity observed in our isolates is consistent with the production of organic acids, hydrogen peroxide, and, compounds widely documented in LAB (Kumari et al., 2021). Lactic acid disrupts pathogen homeostasis, including that of *Listeria monocytogenes* (Liu et al., 2023), while hydrogen peroxide inhibits bacteria lacking oxidative stress defenses (Brudzynski et al., 2011). Bacteriocins, particularly class IIa peptides, are effective against Gram-positive pathogens such as *L. monocytogenes* (Mohanty et al., 2025). LB 95 strain was the only isolate capable of inhibiting *L. monocytogenes* at all temperatures tested, strongly suggesting the production of a bacteriocin-like compound. *E. faecalis* is known to produce enterocins such as enterocin A, P, and B, which display strong anti-*Bacillus cereus* and anti-*Staphylococcus aureus* activity (Choeisoongnern et al., 2023). The broad and temperature-resistant antibacterial activity of LB 95 is consistent with the production of a thermostable enterocin, as previously reported for non-virulent *E. faecalis* strains used in food systems (Abanoz and Kunduhoglu, 2018). The heatmap analysis of antibacterial activity differed markedly among the LB isolates, with LB 92, LB 94, and especially LB 95 showing the strongest inhibition, while LB 91, LB 100, and LB 152 remained weakly active under several conditions. Activity was generally higher at 37 °C, suggesting optimal production of antimicrobial metabolites at this temperature, in agreement with studies on temperature-modulated LAB activity (Upadhyay et al., 2025). Pathogen susceptibility also varied, with *B. subtilis* and *L. monocytogenes* being more sensitive than *E. coli* and *P. aeruginosa*, reflecting known structural differences between Gram-positive and Gram-negative bacteria (Mai-Prochnow et al., 2016). The clustered heatmap confirmed these trends by grouping highly active profiles (LB 95; 40 °C, LB 152; 40 °C) separately from low-activity conditions (LB 94; 25 °C, LB 92; 25 °C), consistent with recent multivariate phenotyping approaches applied to LAB. Overall, LB 95 emerged as the most effective isolate, exhibiting broad and consistent antibacterial activity across all pathogens.

Antibiotic susceptibility is a critical criterion for selecting LAB strains for food applications (Stefanska et al., 2021). The heatmap revealed clear susceptibility clusters among the isolates. All strains displayed uniform sensitivity to β-lactams and macrolides, confirming their safety profile. Uniform resistance to amikacin, cefoxitin, colistin, levofloxacin, and vancomycin reflected intrinsic resistance mechanisms commonly reported in LAB (Monnet, 2017). Strain-specific differences in ciprofloxacin and gentamicin susceptibility suggested genomic variability within *L. plantarum* and *E. faecalis*. These findings reinforce the need for whole-genome sequencing to confirm the absence of mobile resistance genes, particularly in *Enterococcus* spp. (Fatoba et al., 2022). Despite promising results, several limitations must be acknowledged. Antibacterial activity was assessed using crude supernatants, preventing identification of specific inhibitory metabolites. All experiments were conducted *in vitro*; food matrix interactions and storage conditions may alter LAB performance. Identification relied solely on 16S rDNA sequencing, which cannot detect virulence factors or mobile resistance genes. Technological properties such as survival during processing, sensory impact, and stability in food systems were not evaluated. Future studies should include metabolomic profiling, whole-genome sequencing, and application trials in real food matrices. Advances in LAB genomics and metabolite engineering (Chen et al., 2024; Nawaz et al., 2025) offer promising avenues for developing climate-resilient biopreservative cultures.

## 4 Conclusion

This study demonstrated that LAB isolates obtained from Attiéké ferment-maintained growth and antibacterial activity under elevated temperatures, highlighting their relevance as biopreservative candidates in warming food environments. The isolates belonged to *Lactiplantibacillus plantarum* and *Enterococcus faecalis*, two species commonly associated with African fermented foods and known for their robustness under environmental stress. Several strains, particularly LB 92, LB 94, and LB 95, retained inhibitory activity at 40–42 °C and exhibited distinct metabolic responses, including strain-specific patterns of lactic acid and hydrogen peroxide production. LB 95 emerged as the most promising candidate due to its broad and temperature-resistant antibacterial spectrum, high lactic acid production, and phylogenetic proximity to non-virulent reference strains. The combined antimicrobial, enzymatic, and phylogenetic characteristics of these isolates underscore their potential technological value for biopreservation applications. However, further investigations are required to confirm their safety and functional attributes. Whole-genome sequencing is essential for assessing the presence of bacteriocin operons and for excluding mobile virulence or antibiotic resistance determinants, particularly in *Enterococcus* spp. Metabolomic profiling and application trials in real food matrices will also be necessary to identify the specific inhibitory compounds involved and evaluate performance under practical storage and processing conditions. Overall, the results support the potential of Attiéké-derived LAB, particularly LB 95, as climate-resilient bioprotective cultures and provide a foundation for their future development in sustainable food preservation strategies.

## Data Availability

The datasets presented in this study can be found in online repositories. The names of the repository/repositories and accession number(s) can be found at: https://www.ncbi.nlm.nih.gov/, PZ062416; https://www.ncbi.nlm.nih.gov/, PZ062417; https://www.ncbi.nlm.nih.gov/, PZ062418; https://www.ncbi.nlm.nih.gov/, PZ062419; https://www.ncbi.nlm.nih.gov/, PZ062420; https://www.ncbi.nlm.nih.gov/, PZ062421.
